# Multi-functional Ultrasonic Micro-elastography Imaging System

**DOI:** 10.1038/s41598-017-01210-8

**Published:** 2017-04-27

**Authors:** Xuejun Qian, Teng Ma, Mingyue Yu, Xiaoyang Chen, K. Kirk Shung, Qifa Zhou

**Affiliations:** 1 USC Roski Eye Institute, University of Southern California, Los Angeles, CA USA; 2 Department of Biomedical Engineering, University of Southern California, Los Angeles, CA USA

## Abstract

In clinical decision making, in addition to anatomical information, biomechanical properties of soft tissues may provide additional clues for disease diagnosis. Given the fact that most of diseases are originated from micron sized structures, an elastography imaging system of fine resolution (~100 µm) and deep penetration depth capable of providing both qualitative and quantitative measurements of biomechanical properties is desired. Here, we report a newly developed multi-functional ultrasonic micro-elastography imaging system in which acoustic radiation force impulse imaging (ARFI) and shear wave elasticity imaging (SWEI) are implemented. To accomplish this, the 4.5 MHz/40 MHz transducer were used as the excitation/detection source, respectively. The imaging system was tested with tissue-mimicking phantoms and an *ex vivo* chicken liver through 2D/3D imaging. The measured lateral/axial elastography resolution and field of view are 223.7 ± 20.1/109.8 ± 6.9 µm and 1.5 mm for ARFI, 543.6 ± 39.3/117.6 ± 8.7 µm and 2 mm for SWEI, respectively. These results demonstrate that the promising capability of this high resolution elastography imaging system for characterizing tissue biomechanical properties at microscale level and its translational potential into clinical practice.

## Introduction

The biomechanical properties of the soft tissue are highly correlated to pathological and functional change such as the growth of tumor, inflammation and infection^1^. Elastography is a medical imaging modality capable of mapping the biomechanical properties of soft tissue, providing an additional contrast mechanism and clinically relevant information for disease diagnosis and tissue characterization^2, 3^. A typical elastography imaging system is composed of two parts: excitation and detection. The excitation source is responsible for inducing deformation inside the tissue via static compression or dynamic vibration, and the detection part utilizes the traditional imaging techniques, such as ultrasound^4^, magnetic resonance imaging (MRI)^5^ and optical coherence tomography (OCT)^6^, to track the strain or shear wave propagation inside the tissue so as to construct the stiffness map. Thus, the spatial resolution and penetration depth of elastography imaging is synergistically determined by the properties of excitation source and imaging capability of detection source.

In early 1990s, quasistatic ultrasonic elastography has been developed to diagnose the liver fibrosis and breast cancer by measuring the deformation of soft tissue under a manual compression force^7^. However, the diagnostic procedure of quasistatic elastography is highly dependent on the physician’s experience, and its accuracy is distorted by any intervening tissue and an increase in penetration depth. In the recent decade, acoustic radiation force (ARF) has been developed to serve as the remote excitation source to precisely induce a controllable deformation (less than 1%) within targeted region of interest (ROI)^8, 9^. Many ARF-based ultrasonic elastography methods, such as acoustic radiation force impulse (ARFI) imaging^10, 11^, shear wave elasticity imaging (SWEI)^12, 13^, harmonic motion imaging (HMI)^14, 15^ and supersonic shear imaging (SSI)^16, 17^, capitalizing on the advantage of synchronization of ARF excitation and ultrasonic detection, have been used to quantify the mechanical properties of soft tissue in a more effective and accurate manner. However, most of the commercial and research ultrasonic elastography studies, carried out in the standard clinical frequency range, could only provide spatial resolution ranging from sub-millimeter to several millimeters and significantly narrows clinical applications that require microscale level visualization, for example, early stage cancer diagnosis, tumor margin detection, ophthalmologic tissue characterization, and atherosclerotic plaque composition analysis. Optical coherence elastography (OCE), the so-called optical analog of ultrasonic elastography, is a newly developed high resolution elastography technique based on the OCT^18–20^. Within the classification of OCE, acoustic radiation force optical coherence elastography (ARF-OCE)^21^ and ultrasonically-induced shear wave optical coherence elastography^22–24^ are the prominent techniques that combine the dynamic ultrasonic excitation and high resolution optical detection for characterizing the biomechanical properties of soft tissue at the microscale level. However, similar to other optical techniques, the shallow penetration depth significantly limits its translational potential in the clinical study.

To fill the gap between conventional ultrasonic elastography and OCE on spatial resolution and penetration depth, developing a high resolution ultrasonic elastography attracts an increasing interest in both research and clinical studies. To achieve this goal, the novel methodology of low-frequency ARF excitation for effective inducement of tissue deformation, and high frequency detection for accurate quantification of tissue biomechanical properties has been raised. Specifically, Shih *et al*. accomplished ARFI imaging to assess the porcine corneal sclerosis by using a dual-frequency confocal transducer, however, the fixed confocal transducer impairs its capability to access the absolute Young’s modulus and in addition, this method requires axial depth scan to increase its field of view (FOV) at the expense of time^25^. Later, Yeh *et al*. implemented SWEI to monitor the process of mouse liver fibrosis using two side by side single-element transducers; the absolute Young’s modulus at different liver fibrous stages on a large uniform region were measured using shear wave speed reconstruction algorithm^26^. But no 2D/3D elasticity image or spatial resolution were reported in this work. Moreover, no study has shown that ARF-based ultrasound-only elastography achieved a micron-level imaging resolution together with a large FOV.

Herein, we develop a multi-functional ultrasonic micro-elastography imaging system to provide both qualitative (via ARFI) and quantitative (via SWEI) measurements of tissue biomechanical properties on the microscale with Young’s modulus in the range of 0.1–100 kPa. Since the open platform of high frequency ultrasound transducer (>35 MHz) and imaging system are not released, dual frequency single-element transducers, including a 4.5 MHz focused ring shape transducer to generate effective ultrasonic excitation and a 40 MHz unfocused needle transducer to precisely detect tissue deformation, were designed and implemented here to demostrate the concept of ultrasonic micro-elastography. The performance of our high-resolution imaging system was verified on 2D/3D gelatin tissue-mimicking phantoms and an *ex vivo* chicken liver, in which ARFI provides a higher spatial resolution and a faster data acquisition speed, whereas SWEI affords the absolute Young’s modulus and a better image contrast. These results demonstrate that this ultrasonic micro-elastography imaging system is able to provide comparable full-width at half maximum (FWHM) spatial resolution and preferable FOV in comparison with OCE imaging techniques, which indicate its promising future for improving the diagnosis for multiple clinical applications.

## Results

### Acoustic Parameters of Ultrasonic Transducers

A hydrophone (HGL-0085, ONDA Co, Sunnyvale, CA, USA) was used to measure acoustic fields of both excitation transducer and detection transducers, and the acquired data was recorded by an oscilloscope (WaveRunner 104MXi, LeCroy Co, Chestnut Ridge, NY, USA) for off-line analysis.

Based on selected excitation parameters in the actual experiment, I_sppa_ of the excitation transducer at the focal point was 49.01 W/cm^2^ in water condition. Taking into account the derating factor of acoustic attenuation in liver tissue (0.9 dB/cm-MHz), the calibrated I_sppa_ was 36.9 W/cm^2^ and its corresponding I_spta_ was 369 mW/cm^2^. The propagation distance of the excitation transducer (f-number = 1) is 30 mm in axial direction. The measured beam profile has a −6 dB beamwidth and −3 dB depth of focus (DOF) of 350 µm and 2.6 mm, respectively. With respect to the detection transducer, there is a 2 mm axial depth region maintaining 280 µm beamwidth at −3 dB level.

**Figure 1:**
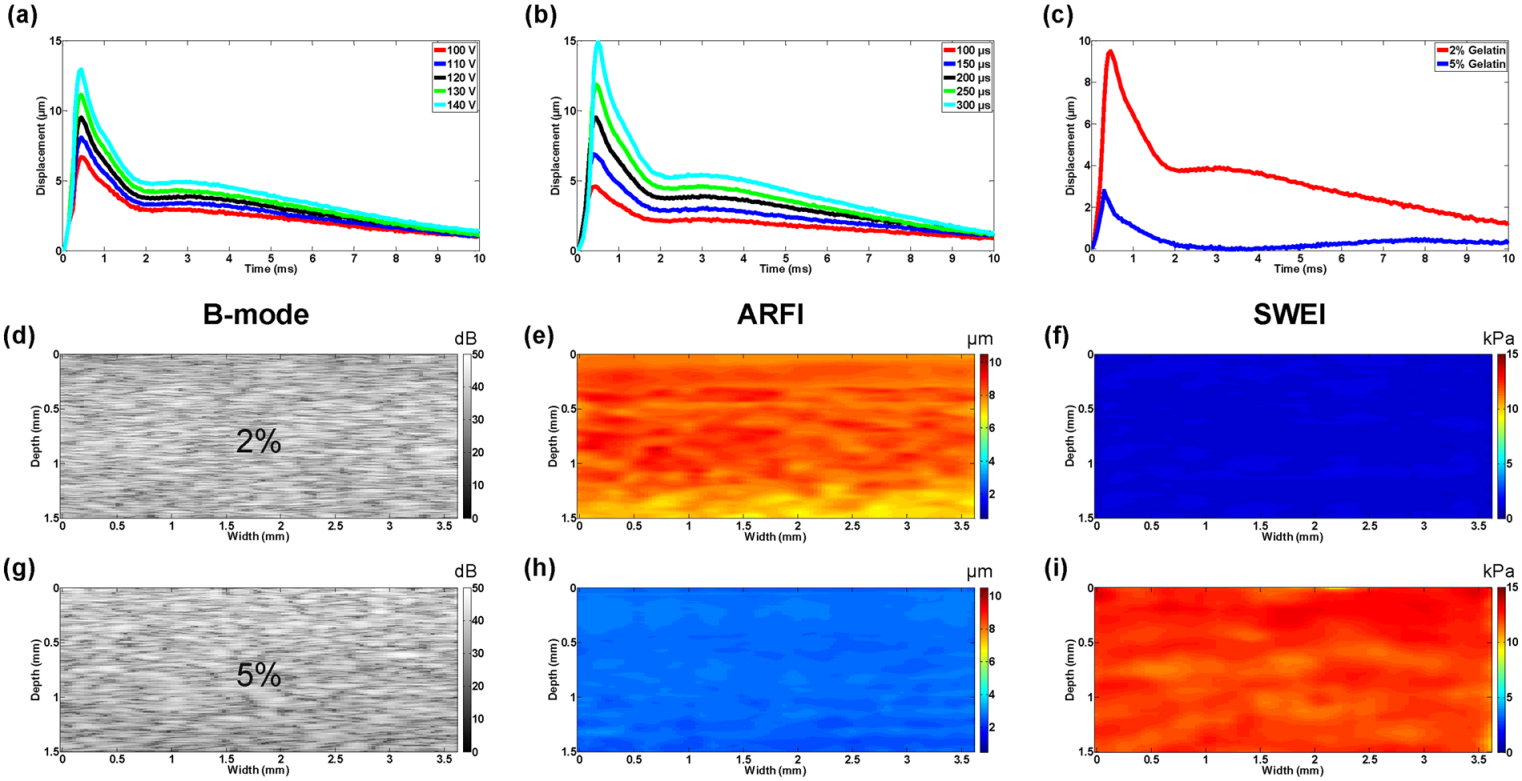
Imaging system validation on homogeneous gelatin phantom. (**a**–**c**) Dynamic displacement curves under different excitation voltage/excitation duration and phantom stiffness. B-mode/ARFI/SWEI images of homogeneous phantom at (**d**–**f**) 2% gelatin concentration, (**g**–**i**) 5% gelatin concentration.

Figure 1(a–c) shows the induced dynamic displacement curves under different excitation voltage, excitation duration and phantom stiffness. Based on our previous experience, the peak displacement should maintain at least 1 µm but less than 10 µm so as to sustain a good SNR while avoiding any potential ultrasound bio-effects, such as thermal effect. It was demonstrated that 120 V and 200 µs were the reasonable excitation parameters in this study.

### Gelatin Tissue-mimicking Phantom Imaging

To avoid ineffective mapping, the FOV of ARFI and SWEI need to be determined before conducting the experiment. Homogeneous gelatin tissue-mimicking phantoms with different stiffness (2% and 5% gelatin concentration) were used to determine the FOV of the imaging system. The effective FOV of ARFI is 1.5 mm in depth indicated by maximum displacement region within 5% discrepancy. The corresponding FOV of SWEI is around 2 mm in depth specified by TOF-based linear regression algorithm with a 0.95 threshold of R^2^ to meet goodness-of-fit metrics. In general, SWEI provides a slightly larger effective FOV than ARFI for the same imaging subject.

The same homogeneous phantoms were next applied to verify the stability of our imaging system by ten times iteration experiment. The repeated experiments showed that ARFI has 8.5 ± 0.5 µm (2% gelatin) and 3.05 ± 0.15 µm (5% gelatin) maximum displacement and SWEI has 1.13 ± 0.07 kPa (2% gelatin) and 12.18 ± 0.42 kPa (5% gelatin) Young’s modulus, respectively. Figure 1(d–i) displays the corresponding imaging results. The same homogeneity was observed in B-mode, ARFI and SWEI images. It was observed that the stiffer phantom had a smaller displacement and a larger Young’s modulus. As expected, it reflects that the displacement in ARFI has the inverse relation with Young’s modulus in SWEI. In color-coded elasticity map, the softer region was mapped to the red color in ARFI and the blue color in SWEI while the stiffer region was mapped to the blue color in ARFI and the red color in SWEI. To check the accuracy of the reconstructed Young’s modulus in SWEI, uniaxial mechanical testing (Model 5942, Instron Corp., MA, USA) was performed on the same phantoms immediately after the experiment. The testing Young’s modulus are 1.26 ± 0.1 kPa and 12 ± 0.5 kPa, respectively. The results show that values in SWEI are slightly different from the values got in the mechanical test.

**Figure 2:**
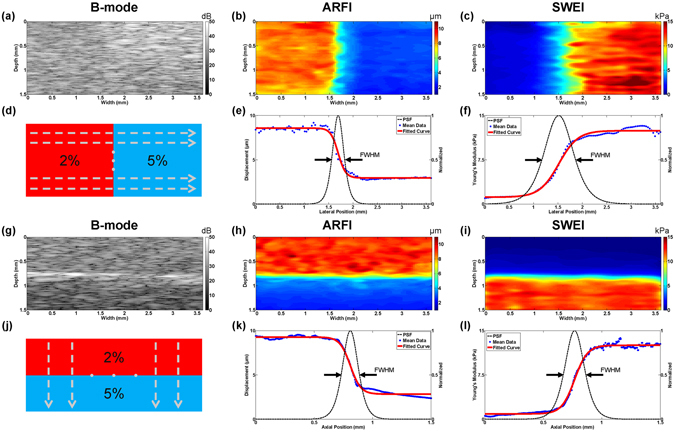
Bi-layer phantom imaging results and its corresponding FWHM resolution. 2D cross-sectional B-mode image, ARFI image and SWEI image. (**a**–**c**) Left-right phantom with a vertical boundary at a width of 1.6 mm; (**g**–**i**) Up-down phantom with a horizontal boundary at a depth of 0.8 mm; (**d**,**j**) Schematic diagram of uniformly selected curve profiles, N = 20 for both Left-right phantom and Up-down phantom; Lateral FWHM resolution model of (**e**) ARFI image and (**f**) SWEI image; Axial FWHM resolution model of (**k**) ARFI image and (**l**) SWEI image.

Figure 2(a–c,g–i) displays B-mode images and the corresponding ARFI/SWEI images of inhomogeneous bi-layer phantoms. It was observed that the regions with different stiffness exhibited a homogeneous echogenicity in the B-mode image and less contrast information (except for some bright speckle signals caused by the precipitation of silicon dioxide powder in up-down phantom). However, in ARFI image, the relative stiffness distribution could be easily distinguished and its border was clearly visualized from the sharp transition. In SWEI image, the mapping of the absolute Young’s modulus exhibited a relative broad transition edge, especially for lateral direction. Outside transition region, it was observed that the Young’s modulus in each layer were well matched with the measured Young’s modulus in corresponding homogeneous phantom indicated by the similar color under the same color bar.

**Figure 3:**
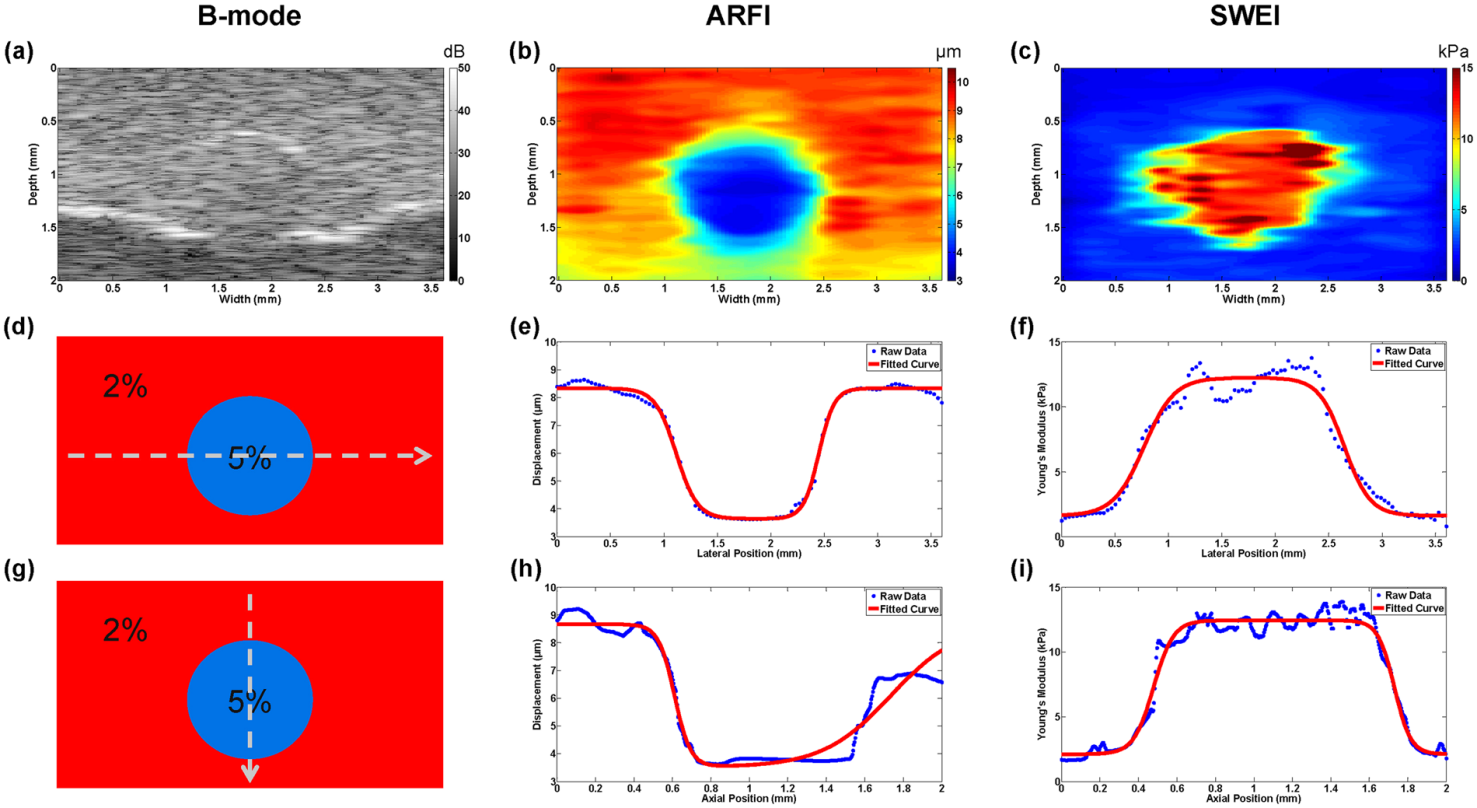
Inclusion phantom imaging results and transitional curve profiles. (**a**–**c**) 2D cross-sectional B-mode image, ARFI image and SWEI image. The data profiles (**d**) at a depth of 1.1 mm and (**g**) at a width of 1.8 mm were selected. The raw data and fitted curve of ARFI and SWEI were plotted along (**e**,**f**) lateral direction, and (**h**,**i**) axial direction. The phantom has a 1.2 mm diameter cylindrical inclusion with a background-interior-background structure.

The cross sectional image results of the phantom with a cylindrical inclusion are shown in Fig. 3(a–c). Both B-mode and ARFI image have a clear circular-shape boundary in good agreement with designed morphological feature. SWEI image shows an irregular circular-shape with a bulge in deformation due to the poor elastography resolution. Besides structure information, the relative/absolute stiffness distribution was clearly delineated in ARFI/SWEI. However, it was observed that the deeper image region of ARFI had a significantly reduced peak displacement while SWEI image maintained a uniform stiffness distribution beyond 1.5 mm. This phenomenon was caused by the different effective FOV of ARFI and SWEI. It was indicated that time-to-peak (TTP) displacement may be expected to be independent of acoustic attenuation within the FOV while maximum displacement still suffers from acoustic attenuation within the FOV, especially for deeper region^27^.

### Spatial Resolution and Image Contrast

High spatial resolution elastography is needed to image soft tissues at a small scale, especially in the micron range. To evaluate the performance of our imaging system, bi-layer phantoms with vertical/horizontal transition edge were used to quantify the lateral/axial resolution of ARFI and SWEI, respectively. The edge spread function (ESF) can be obtained by fitting the experimentally measured curve profile into a sigmoid function model using the following equation (1)

1 $$p({x})=({{p}}_{1}-{{p}}_{2})[\frac{1}{{e}^{-(x-b)/\lambda }+1}]+{{p}}_{2}$$

where *x* is the lateral position, *p*_1_ and *p*_2_ represent displacement in ARFI and Young’s modulus in SWEI, respectively. *b* is the location of the layer boundary, *λ* represents the width of the ESF - transition from one layer to another. Giving a curve profile, all parameters in the sigmoid function model were estimated using standard non-linear least squares fitting algorithm^28^. The first derivative of ESF results in a point spread function (PSF). Then the FWHM of the PSF is used to estimate elastography resolution^29, 30^. The mathematical formula of FWHM resolution is given in equation (2).

2 $${\rm{FWHM}}=2\,\mathrm{ln}(3+2\sqrt{2})\lambda $$

Figure 2(e,f,k,l) shows the FWHM resolution calculated from the sigmoid fitting model in the lateral and axial direction of ARFI and SWEI. For statistical analysis, 20 horizontal/vertical experimental curve profiles selected uniformly from bi-layer phantoms shown in Fig. 2(d,j) were used to calculate FWHM resolution distribution. Finally, the averaged FWHM value was treated as the lateral/axial resolution of actual elastography image. The measured lateral and axial resolutions are 223.7 ± 20.1 µm and 109.8 ± 6.9 µm for ARFI, 543.6 ± 39.3 µm and 117.6 ± 8.7 µm for SWEI, respectively.

Inclusion phantom with background-interior-background profiles in all direction represents a complex multi-layer structure. Because there are two transition edges in each direction, equation (1) was rewritten as the product of two sigmoid functions. To observe the lateral/axial resolution of inclusion phantom, raw curve profiles were selected across through the center of inclusion at a depth of 1.1 mm and at a width of 1.8 mm, respectively. Figure 3(d,g) shows the diagram of selected curves. Figure 3(e,f,h,i) displays the raw data profiles and fitting results of inclusion phantom along the lateral and axial direction. These symmetrical curves are well matched with the symmetric property of circular inclusion, except the one in Fig. 3(h) which was caused by attenuated displacement beyond 1.5 mm in ARFI. It was indicated that there is a wider transition width in inclusion phantom than that in bi-layer phantom due to the non-prefect flat surface.

In addition to spatial resolution, image quality is also assessed by contrast. The previous study shows that image contrast is affected by tissue stiffness difference and the relation between region of excitation (ROE) size and the volume of the lesion^31^. The equation (3) is used to evaluate the contrast of ARFI and SWEI on the inclusion phantom

3 $${Contrast}=\,\frac{|{P}_{in}-{P}_{out}|}{{P}_{out}}$$

where *P*_*in*_ and *P*_*out*_ are the mean pixel values of the target and background, respectively. The calculated contrast is 0.59 for ARFI and 7.66 for SWEI.

### 3D Imaging of Tissue-mimicking Phantom and Chicken Liver

Medicine, biology, and many other areas have been revolutionized by 3D imaging techniques. In the medical imaging field, 3D images contain much more valuable information that 2D images cannot readily provide, such as the accurate position, volume of the target of interest, and tissue anisotropy. Therefore, 3D imaging helps physicians perform interventions in a more convenient way. The 3D imaging capability of our imaging system was verified with a side-by-side gelatin phantom and an *ex vivo* chicken liver. 2D mechanical scanning was conducted to reconstruct 3D images. The volume dimensions (xyz) are 1.8 × 3.6 × 2 mm and 1.2 × 2 × 1.8 mm for phantom and chicken liver, respectively.

**Figure 4:**
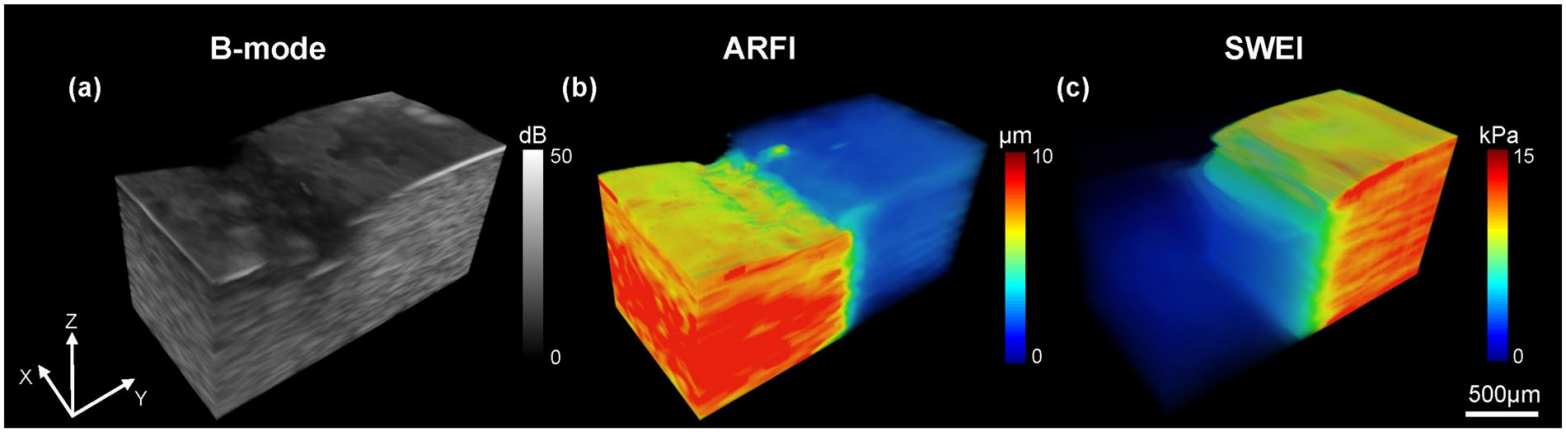
3D visualizations of a side by side gelatin tissue-mimicking phantom. (**a**) B-mode image, (**b**) ARFI image and (**c**) SWEI image. Volume dimensions (xyz) are 1.8 mm × 3.6 mm × 2 mm.

Figure 4 portrays a 3D B-mode image to observe a stereo morphological structure of phantom side by side and the corresponding ARFI/SWEI images to view the biomechanical properties distribution. In Fig. 4(a), the B-mode image provides 3D structure information and shows little contrast for left-right transition. The high resolution ARFI image shows a highly correlated morphological structure with the B-mode image, especially for the irregular shape of surface in Fig. 4(b). In addition, 3D stiffness distribution supplies a good contrast for the left-right transition. In SWEI image, although it does not maintain the same structure compared with ARFI, a better image contrast together with the absolute Young’s modulus is a great advantage over ARFI. For example, a 2D transition surface is clearly displayed in Fig. 4(c).

**Figure 5:**
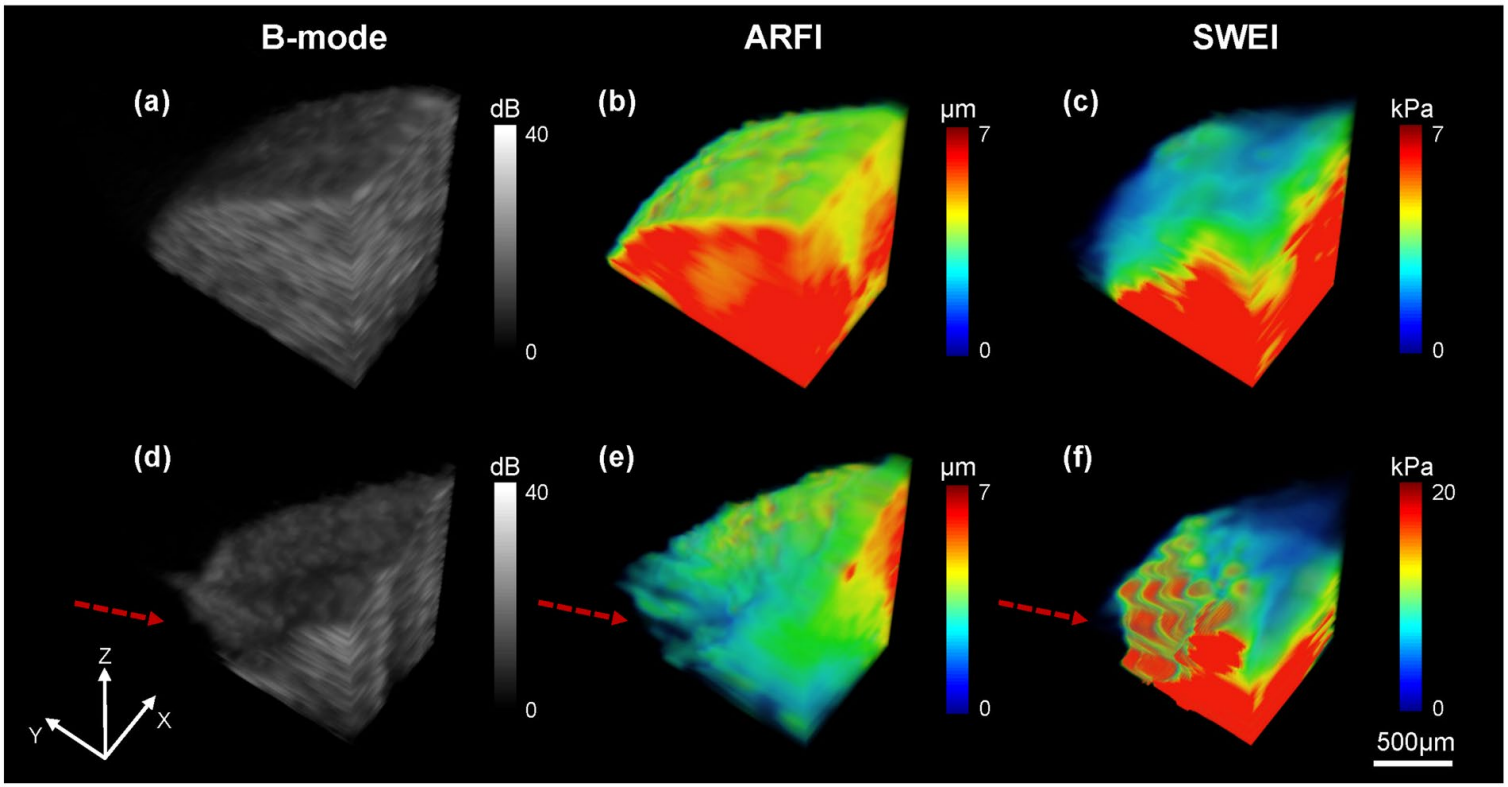
*Ex vivo* 3D visualizations of the chicken liver. (**a**–**c**) Normal liver and (**d**–**f**) Liver with artificially induced “fibrosis” using formalin solution. Volume dimensions (xyz) are 1.2 mm × 2 mm × 1.8 mm. The red arrows in (**d–f**) indicate the site of formalin injection.

Figure 5(a,d) show the B-mode images of normal and formalin-fixed chicken liver, respectively. The small anatomical structures of the chicken liver were clearly revealed in the B-mode image where the morphological difference between normal and formalin fixed liver was caused by injection operation. The corresponding ARFI images in Fig. 5(b,e) point out that the normal liver tends to have a homogeneous stiffness distribution indicated by the same color and the formalin-fixed region located at the left bottom area of the liver has a smaller displacement with a gradually changing color. The high resolution of ARFI image provides a well-matched morphological structure with the B-mode image, especially for the uneven surface of the formalin-fixed liver. Moreover, an accurate stiffness distribution and the diffusion process of “fibrosis” were observed in ARFI image. SWEI image results are shown in Fig. 5(c,f). The average reconstructed Young’s modulus for the normal liver is 5.69 ± 0.8 kPa which is smaller than 5.8 ± 0.35 kPa performed by the standard uniaxial mechanical testing. In the formalin-fixed region, it was observed that there is a significant increasing stiffness with the average measured Young’s modulus −19.22 ± 2.2 kPa in SWEI image. Clearly, the spatial resolution of ARFI is better than SWEI, particularly for the serious distorted formalin-fixed liver. However, SWEI has a better contrast indicated by a larger dynamic range color bar.

## Discussions

The output power of excitation transducer was carefully studied. Both of I_sppa_ and I_spta_ were within the limitation of United States Food and Drug Administration (FDA). For single short exposure time (less than 1 ms), the output power was much lower than the threshold of occurring ultrasound bio-effects. In the actual experiment, the beamwidth of excitation transducer was larger than motor step size, which means that each scanning position may receive multiple excitation force from adjacent positions. However, the time interval to receive multiple excitation force also extends. Thus, the temporal average intensity still not exceed the limitation of FDA and has a small risk to increase the temperature level.

The measured DOF of excitation transducer is 2.6 mm, and the detection transducer has a 2 mm DOF with a nearly uniform beamwidth. The elastography imaging resolution was guaranteed by imaging our target within the maximum overlap of the two DOFs which is the definition of elastography FOV. It was well-documented that the spatial resolution of elastography is determined by both excitation and detection transducers as well as signal processing parameters. Specifically, axial resolution is on the order of ultrasonic wavelength and lateral resolution is proportional to the beamwidth of ultrasonic system (an empirical expression of elastographic lateral resolution is given by ${R}_{l}=k\cdot d$, where *d* is the beamwidth of ultrasonic system, the value of *k* would not exceed 1)^32, 33^. Righetti *et al*. performed a simulation study to estimate the spatial resolution of elastography based on the distance between the FWHM of the strain profiles of two equally stiff lesions embedded in a softer homogeneous background^32, 33^. However, fabricating a phantom with a very thin transition layer separated by two equal lesions is very challenging, especially for the high resolution imaging such as optical imaging and high frequency ultrasonic imaging. In addition, mechanical parameters such as the contrast-transfer efficiency and complex boundary conditions will affect the practically achievable resolution^34^. Therefore, a simplified bi-layer phantom with distinguished stiffness transition in horizontal/vertical direction has been used to experimentally quantify imaging resolution of high frequency ultrasound and OCT^18, 25^. In 2012, Rouze *et al*. developed a sigmoid function fitting model method and define 20–80% transition width as elastography resolution^28^. However, “20–80% transition” resolution, as a relative parameter to evaluate resolution performance, cannot exactly represent the resolution of actual elastography images. PSF, the first derivative of ESF, describing the impulse response of an imaging system seems to be a more standard approach. Therefore, the spatial resolution of elastography was defined as the “FWHM of the PSF” in this study.

Compared with ARFI, the lateral resolution of SWEI is additionally restricted by off-axis distance (Δ*x*) due to boundary conditions. In Fig. 2(b,c), it is noticed that the width of transition boundary of ARFI is sharper than SWEI because a propagation distance is needed for time-of-flight (TOF) shear wave speed (SWS) reconstruction algorithm and SWS is likely misleading by reflection or refraction at the boundary. In Fig. 5(f), the distorted morphological structure and some estimation artifacts were observed in the *ex vivo* formalin-fixed chicken liver. Due to the reconstruction inaccuracy, the boundary exhibits a wide transition region, failing to resolve the exact edge position in SWEI. Therefore,  the lateral resolution of SWEI will be greatly influenced by its propagation distance (Δ*x* = 350 μm) and the excitation beam width, which is much worse than that of ARFI. The axial resolution of SWEI is comparable with that of ARFI. The possible reason is that the horizontal edge is paralleling to the shear wave propagation direction in Fig. 2(i) where less shear wave distortion phenomenon occurs.

Apparently, attenuation of ARF varies from tissue to tissue. Cook *et al*. indicated that acoustic attenuation of gelatin tissue-mimicking phantom depends on both gelatin and scatter concentration^35^. Based on equation (4) where *k* is larger than 1 by assuming *E*_1_ is greater than *E*_2_, the contrast of SWEI should be better than that of the ARFI theoretically, which was verified by our experimental results. All results demonstrated that ARFI mapping with maximum displacement has a better spatial resolution while SWEI mapping with Young’s modulus obtains a better image contrast.

The results show that the estimated Young’s modulus in SWEI has a small variance compared to that in mechanical testing. The underestimation/overestimation issue was caused by the error of TOF SWS reconstruction. When the shear wave propagation time is less than 50 µs, the TTP displacement timing will be set to either forward timing or backward timing limited by 20 kHz PRF. Although the PRF was increased to 60 kHz with spline interpolation to track the sub-timing position to reduce the error in this study, the measured propagation time is still slightly different from the true propagation time. Besides, the SNR of induced peak displacement in SWEI is lower than that in ARFI because all detection positions are out of ROE, which leads to some artifacts in TOF SWS reconstruction results. Moreover, the liver tissue is not a pure elastic and isotropic medium, especially for the formalin-fixed liver^36^. Shear wave dispersion and irregular structure may also reduce the accuracy of TOF SWS reconstruction result. Thus, a higher PRF and a better SNR are required to further increase the accuracy.

It was well-established that the conventional ultrasound possesses a deep penetration depth while OCT has superior resolution for accessing the anatomy of tissue. The most recent developed ultrahigh resolution OCE achieved spatial resolution less than 2 µm which is highest report to date in optical elastography^37^. Besides the spatial resolution, OCE provides a better sensitivity than ultrasound. The non-contact 4D OCE imaging system^23^ has the ability to detect tissue deformation in nanometer scale while ultrasound is only sensitive to sub-micron or micrometer level deformation. Although swept-source OCT has achieved extended imaging depth in ocular tissue owing to its low sensitivity roll-off with depth^38^, optical imaging is somewhat organ dependent and is still feeble to most highly-scattered tissues other than the eye. Instead, high frequency ultrasound is able to provide a more effective and uniform FOV at deeper tissue while maintaining high spatial resolution, which serves as the desirable imaging technique for various clinical applications. Therefore, our newly developed high resolution ultrasonic micro-elastograhy system was proved to bridge the gap in between the conventional ultrasonic elastography and OCE on spatial resolution and penetration depth.

In summary, both high resolution ARFI and SWEI are essentially useful to characterize tissue biomechanical properties. The experimental results suggest that the multi-functional ultrasonic micro-elastography imaging system provides a non-invasive way to differentiate tissue biomechanical properties with a very fine resolution (~100 µm) and deep penetration depth, indicating the capability to quantify the relative and absolute Young’s modulus using single system setup. Combined 3D morphological and biomechanical information of soft tissue will improve the visualization of the anatomical structure of soft tissues and offer potential diagnostic advantages in clinical application. Although single-element transducers were used in this study, we believe that ultrasonic micro-elastography will have a favorable prospect. The future development of ultrasonic micro-elastography will lie in two aspects: the first aspect is to develop and improve high frequency ultrasound array and data acquisition system. For example, a dual-frequency co-linear array that allows for the electronic steering of both excitation and detection beams is a favorable solution for improving elastography resolution, FOV and frame rate, and such array is currently under development^39^. In the meanwhile, the implantation of the advanced beamforming techniques^40^ for array transducer that have been reported and evaluated in the conventional frequency range, such as plane wave imaging and virtual source imaging, will further improve the performance of ultrasonic micro-elastography techniques. Another aspect is that the small needle transducer can potentially be modified into a handheld device so as to facilitate the tissue differentiation and boundary detection for various deep organ biopsy guidance application including liver. We are confident that the future developed array-based ultrasonic micro-elastography would be a promising technique in both research and clinical studies.

## Methods

### Ultrasonic Micro-elastography Imaging System and Data Acquisition

**Figure 6:**
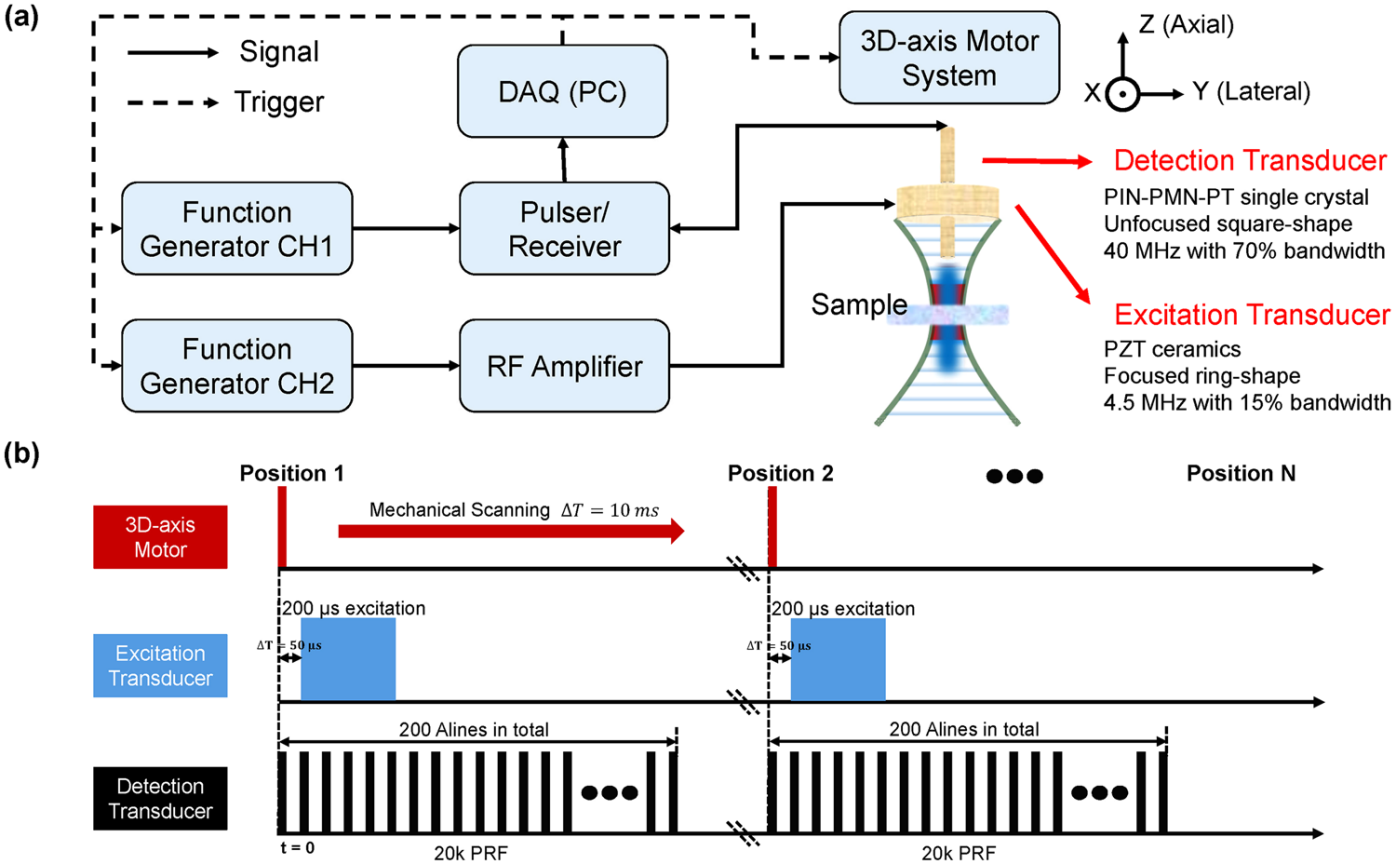
Schematic diagram of the designed multi-functional ultrasonic micro-elastography imaging system. (**a**) The experimental setup and transducer parameters. (**b**) The synchronized timing sequence controlled by multi-functional ultrasonic micro-elastography imaging system.

In order to achieve micro-resolution elastography, a 4.5 MHz ring excitation and a 40 MHz needle detection transducer were carefully designed and fabricated in this study. 4.5 MHz frequency was selected as a proper balance between acoustic intensity and potential bio-effects while 40 MHz needle transducer was used to provide a good spatial resolution. To perform the experiment, the 40 MHz needle detection transducer was first inserted into the center hole of the 4.5 MHz excitation transducer and then two transducers were carefully aligned axially under the guidance of a hydrophone. The schematic diagram of the multi-functional ultrasonic micro-elastography imaging system is shown in Fig. 6(a). To induce tissue motion, the 4.5 MHz excitation transducer was driven by an arbitrary function generator (AFG 3252 C, Tektronix, Beaverton, OR, USA) using 4.5 MHz sinusoid tone bursts with a duration from 100 µs to 300 µs and then amplified between 100 V and 140 V by an RF power amplifier (100A250A, Amplifier Research, Souderton, PA, USA). The 40 MHz needle transducer was driven by a pulser/receiver (JSR500, Ultrasonics, NY, USA) and triggered by the arbitrary function generator with a pulse repetition frequency (PRF) of 20 kHz. Before acquired using a 12-bit digitizer card (ATS9360, Alazartech, Montreal, QC, Canada) at a sampling rate of 1.8 GHz, the ultrasonic signals were filtered by an analog band-pass filter to remove signal contamination of the excitation beam. In order to reduce system jitter, the same clock was used to synchronize the digitizer, pulser/receiver, and arbitrary function generator. Schematic diagram of the synchronized timing sequence is shown in Fig. 6(b). At each scanning position, the radio-frequency (RF) signal was acquired for 10 ms to ensure that the tissue returned to its original position and the excitation transducer was excited 50 µs after the acquisition. All raw RF data were saved to disk for offline processing.

The first tracking A-line at each scanning position served as the reference for the initial tissue position. Then tissue displacements were calculated using 1-D normalized cross-correlation technique with a symmetric search region and 1.5 λ window size^41, 42^. The dynamic displacement data were displayed as the maximum displacement in ARFI and TTP displacement in SWEI^28^. To obtain a 2D/3D image, both of the excitation and detection transducer were mounted on a 3-D stepper motor (SGSP33-200, OptoSigma Corporation, Santa Ana, CA, USA) for mechanical scanning with an increment of 36 μm. Since the data acquisition time at each scanning position is very short (millisecond level), the image reconstruction rate is mainly determined by motor speed and selected scanning distance. In this study, few seconds are required to reconstruct a 2D image which is acceptable in the preclinical study. In addition, the detection transducer was placed on another 3-D linear stage to control the distance between excitation and detection transducer. The final 2D/3D images were obtained after a median filter in order to increase the signal to noise ratio (SNR). Data collection and analysis process were performed using MATLAB (The MathWorks, Natick, MA, USA).

### Imaging Subjects Preparation

The gelatin (Gelatin G8-500, Fisher Scientific, USA) based tissue-mimicking phantoms with the same concentration of silicon carbide powder (S5631, Sigma-Aldrich, St.Louis, MO, USA) as the sound scatters were fabricated. Phantoms comprising gelatin at concentrations of 2% and 5% were used in this study to represent materials of different stiffness^43^. All phantoms have a cylindrical shape with a 50 mm diameter and 12 mm height. The homogeneous phantoms with 2% gelatin concentration and 5% gelatin concentration were used to build the calibration data. Bi-layer Phantom consisting of 2% gelatin concentration on the left and 5% on the right were used to calibrate lateral resolution. Bi-layer phantom consisting of 2% gelatin concentration on the top and 5% at the bottom were used to calibrate axial resolution. The phantom with a 1.2 mm diameter 5% gelatin concentration cylindrical inclusion surrounded by 2% gelatin concentration background was used to test the feasibility of our high resolution imaging system on a more complex structure.

Liver tissue was selected as an imaging subject because of the following reasons. First, the effectiveness of some newly developed drugs or therapeutic procedures has to be verified on the small animal model in preclinical studies. Various studies about the progression of liver fibrosis and the evaluation of anti-fibrosis medication have been carried out by performing quantitative imaging of experimental animal^36^. Second, several high resolution elastography imaging techniques^44^ have been reported to be integrated with Fine Needle Aspiration Cytology (FNAC) needle, which enables the future *in vivo* guiding percutaneous biopsy and therapeutic methodologies of the thyroid gland, breast and liver cancer. Ultrasonic micro-elastography can potentially be modified into a handheld device so as to facilitate the tissue differentiation and boundary detection for various deep organ biopsy guidance application including liver.

All chicken livers were bought from a local slaughterhouses (Sierra Medical Science, Inc., Whittier, CA, USA). Experimental procedures were performed in accordance with the guidelines of University of Southern California’s Institutional Animal Care and Use Committee. Both normal and formalin-fixed livers were imaged in this study. Because the increased liver stiffness is linked with the development of fibrosis stage^45^, formalin solution (F79-1 Formaldehyde, Fisher Scientific, Waltham, MA, USA) was used to induce liver cirrhosis to represent the “fibrosis” stage. Chicken liver was immersed with HBSS (Life Technologies Co, CA, USA) solution at room temperature during the experiment.

### ARFI imaging and SWEI imaging

**Figure 7:**
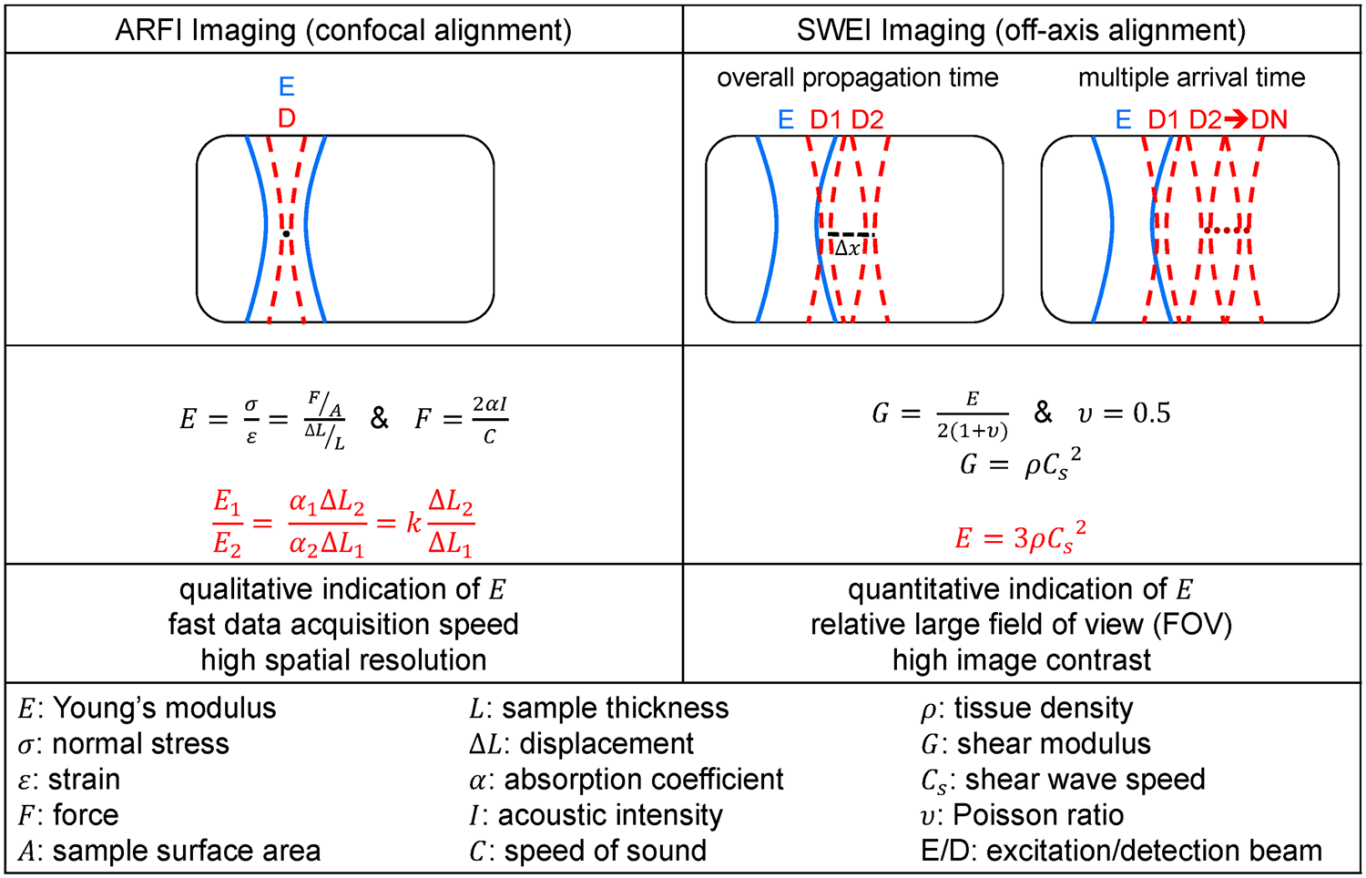
The principle comparison and analysis of ARFI and SWEI.

The multi-functional ultrasonic micro-elastography imaging system provides the capability of characterizing the tissue biomechanical properties using multiple imaging methods, including ARFI and SWEI. The principle comparison and analysis are displayed in Fig. 7. ARFI is a strain imaging method mapping tissue mechanical properties with maximum displacement using equation (4)

4 $$\frac{{E}_{1}}{{E}_{2}}=k\frac{{\rm{\Delta }}{L}_{2}}{{\rm{\Delta }}{L}_{1}}$$

where *E* is Young’s modulus, Δ*L* is the tissue displacement, *k* is determined by the ratio of acoustic attenuation in two different samples^35, 46^. Under the assumption of a purely elastic, incompressible, homogeneous medium, SWEI is used to quantify tissue stiffness by a well-known equation (5)

5 $$E=3\rho {{C}_{s}}^{2}$$

where *E* is the Young’s modulus, *ρ* is the tissue density, *C*_*s*_ is the shear wave speed. By further assuming a fixed direction of propagation (perpendicular to excitation beam axis) and negligible dispersion issue, the SWS can be calculated from the TOF based approach^47, 48^. In TOF SWS reconstruction algorithm, the shear wave propagation timing at each lateral off-axis position outside the ROE is indicated by shear wave’s peak displacement. Giving a lateral propagation distance (Δ*x*), TOF SWS can be calculated by overall propagation time (two detection positions) approach using ${C}_{s}=\frac{{\rm{\Delta }}x}{{\rm{\Delta }}t}$ or multiple arrival time (multiple detection positions) approach using linear regression algorithm. Compared with only one detection position of ARFI, the data acquisition time of SWEI is significant longer. Multiple arrival time approach is typically implemented in an array system to image a large region. To get a fine spatial resolution and speed up the data acquisition speed, overall propagation time approach was implemented in this study for SWEI. As indicated at the beginning, the purpose of our imaging system is to characterize small scale soft tissues with expected Young’s modulus in the range 0.1–100 kPa. By assuming a constant density of soft tissue, typically 1000 kg/m^3^, the maximum SWS is 5.8 m/s in SWEI. Time interval of TTP displacement profile should be at least 50 µs limited by PRF; therefore, the minimum off-axis distance is 290 µm. In order to satisfy the requirement that the region in which the shear wave propagates is homogeneous and to sustain a high resolution, a small propagation distance is preferred. However, the propagation distance is better to be larger than the beamwidth of the excitation transducer^49^. Thus, a 350 µm laterally off-axis distance (Δ*x*) was used to quantify the SWS with a good balance between precision and resolution.
